# A Hox complex activates and potentiates the Epidermal Growth Factor signaling pathway to specify *Drosophila* oenocytes

**DOI:** 10.1371/journal.pgen.1006910

**Published:** 2017-07-17

**Authors:** Guolun Wang, Lisa Gutzwiller, David Li-Kroeger, Brian Gebelein

**Affiliations:** Division of Developmental Biology, Cincinnati Children’s Hospital, MLC 7007, Cincinnati, OH, United States of America; New York University, UNITED STATES

## Abstract

Hox transcription factors specify distinct cell types along the anterior-posterior axis of metazoans by regulating target genes that modulate signaling pathways. A well-established example is the induction of Epidermal Growth Factor (EGF) signaling by an Abdominal-A (Abd-A) Hox complex during the specification of *Drosophila* hepatocyte-like cells (oenocytes). Previous studies revealed that Abd-A is non-cell autonomously required to promote oenocyte fate by directly activating a gene (*rhomboid*) that triggers EGF secretion from sensory organ precursor (SOP) cells. Neighboring cells that receive the EGF signal initiate a largely unknown pathway to promote oenocyte fate. Here, we show that Abd-A also plays a cell autonomous role in inducing oenocyte fate by activating the expression of the Pointed-P1 (PntP1) ETS transcription factor downstream of EGF signaling. Genetic studies demonstrate that both PntP1 and PntP2 are required for oenocyte specification. Moreover, we found that PntP1 contains a conserved enhancer (*PntP1OE*) that is activated in oenocyte precursor cells by EGF signaling via direct regulation by the Pnt transcription factors as well as a transcription factor complex consisting of Abd-A, Extradenticle, and Homothorax. Our findings demonstrate that the same Abd-A Hox complex required for sending the EGF signal from SOP cells, enhances the competency of receiving cells to select oenocyte cell fate by up-regulating PntP1. Since PntP1 is a downstream effector of EGF signaling, these findings provide insight into how a Hox factor can both trigger and potentiate the EGF signal to promote an essential cell fate along the body plan.

## Author summary

*Hox* genes encode a conserved family of transcription factors that regulate unique cell types within the distinct morphological structures that arise along the body plan of animals. Hox transcription factors specify different cell fates by regulating the expression of downstream genes, many of which are part of cell signaling pathways. For example, a Hox factor that is only expressed in the *Drosophila* abdomen (Abdominal-A) activates the release of a signaling molecule (Epidermal Growth Factor, EGF) from a specific neural precursor cell. The cells that receive the signal are induced to become essential hepatocyte-like cells required for metabolism and animal growth. Here, we show that this same Hox factor is not only required for sending the EGF signal, but it also enhances the strength of the signal within the developing hepatocyte-like cells. Importantly, the thoracic Hox factor fails to both induce and enhance the signal, thereby providing a better understanding of how these abdomen-specific cells are generated and distinct morphological structures become regionalized to specific segments of the embryo.

## Introduction

Hox genes encode a family of conserved homeodomain transcription factors that pattern the developing anterior-posterior (A-P) axis of metazoans by regulating target gene expression [1–5]. Most animals contain five or more Hox genes with each being expressed in distinct A-P regions (i.e. segments) to specify distinct morphological structures and cell fates in organs such as the nervous system, musculature, digestive system, and appendages [6, 7]. Developmental genetic and genomic studies have revealed that Hox factors interact with additional morphogenetic pathways to specify diverse cell types via the regulation of numerous downstream target genes [8–11]. Ultimately, these target genes contribute to gene regulatory networks (GRNs) that ensure appropriate cell fate specification within different organ systems [12]. Understanding how Hox factors intersect with other growth control pathways is therefore likely to reveal fundamental insight into both animal development and evolution.

Functional studies in organisms from worms to mice have revealed that the integration of Hox factors with signaling pathways is a common mechanism used for the specification of distinct cell types. Examples include the integration of Wnt, Epidermal Growth Factor (EGF) and Notch signals with Hox factors during the specification of different epidermal and vulval fates in *C*. *elegans* [13, 14], the integration of retinoic acid (RA) and Fibroblast Growth Factor (FGF) signaling with Hox factors during spinal cord and hindbrain development in vertebrates [15–18], and the integration of the Jak/Stat signaling pathway with the Abdominal-B (Abd-B) Hox factor during the formation of the *Drosophila* genitalia [19, 20]. While the gene regulatory networks underlying Hox-mediated cell type specification are just beginning to be defined, these studies emphasize that synergy between Hox factors and growth factor signaling pathways are frequently required for proper organogenesis.

The development of larval oenocytes in the *Drosophila* embryo represents a well-characterized example of how a Hox factor (Abdominal-A, Abd-A) specifies a segment-specific cell type by directly regulating a signaling pathway [21, 22]. Larval oenocytes are hepatocyte-like cells that regulate lipid and nutrient metabolism required for animal growth [23]. Together with the cells of the fat body, larval oenocytes perform many of the functions of the mammalian liver. Larval oenocytes are specified in clusters of three to nine cells from the dorsal ectoderm of seven abdominal segments (A1 through A7) during *Drosophila* embryogenesis [22]. Developmental genetic studies have uncovered that the Epidermal Growth Factor (EGF) pathway, the Spalt transcription factors, and the Abdominal-A (Abd-A) Hox factor as well as the Extradenticle (Exd) and Homothorax (Hth) Hox co-factors are all required for proper oenocyte development [22, 24–26]. The current model for oenocyte specification is that an Abd-A/Hth/Exd transcription factor complex induces the expression of the *rhomboid* (*rho*) gene in a specific subset of abdominal sensory organ precursor cells (SOPs) [27–29]. *rho* encodes a serine protease that cleaves and promotes the secretion of the Spitz (Spi) EGF ligand, and the neighboring ectoderm cells of the dorsal ectoderm, which express the Spalt-family of zinc finger transcription factors (Spalt-major (Salm) and Spalt-related (Salr)), receive the EGF ligand, further up-regulate the Spalt transcription factors, and initiate a largely unknown genetic cascade that commits the cell to an oenocyte fate [24, 25, 30, 31]. Other Hox factors fail to activate the *rho* protease in SOP cells, and thus the EGF signal and subsequent specification of oenocyte fate is restricted to Abd-A-positive segments.

The above model suggests that Abd-A, Exd, and Hth are non-cell autonomously required to specify oenocytes via the activation of *rho* expression and thereby stimulate EGF secretion to promote larval oenocyte development. Consistent with this idea, previous studies have shown that ectopic stimulation of the EGF pathway in thoracic segments, which do not express Abd-A, is sufficient to induce oenocytes [22]. However, in this study, we show that Abd-A also plays a positive role within oenocyte precursors by potentiating the EGF signal. First, we used genetics to demonstrate that the two ETS transcription factors encoded by *pointed* (*pnt*) (*pntP1* and *pntP2*) are each required for oenocyte specification. Both Pnt factors, which are produced via alternative promoters, are activated by EGF signaling albeit via distinct mechanisms [32–36]. PntP2 requires direct phosphorylation by MAPK to activate transcription, whereas PntP1 is transcriptionally up-regulated by the EGF pathway through largely unknown mechanisms [37]. Second, we identified a PntP1 specific *cis*-regulatory module (CRM) that is activated in oenocytes via direct regulation by an Abd-A/Exd/Hth Hox complex. In addition, we found that this PntP1 enhancer (*PntP1OE*) is stimulated by the EGF pathway and contains ETS binding sites required for optimal enhancer activation. These findings support a model in which a transient EGF signal phosphorylates and activates the PntP2 transcription factor, and PntP2, in parallel with an Abd-A Hox complex, directly activates *PntP1OE* transcription in oenocyte precursor cells. Importantly, unlike PntP2, the PntP1 transcription factor is constitutively active and does not require MAPK phosphorylation to activate gene expression [32, 36, 37]. Thus, Abd-A not only directly stimulates EGF secretion from neighboring abdominal SOP cells, but also potentiates the EGF signal by stimulating PntP1 expression within oenocyte precursor cells.

## Results

### The Pointed-P1 and Pointed-P2 ETS factors are both required for the specification of larval oenocytes

**Fig 1:**
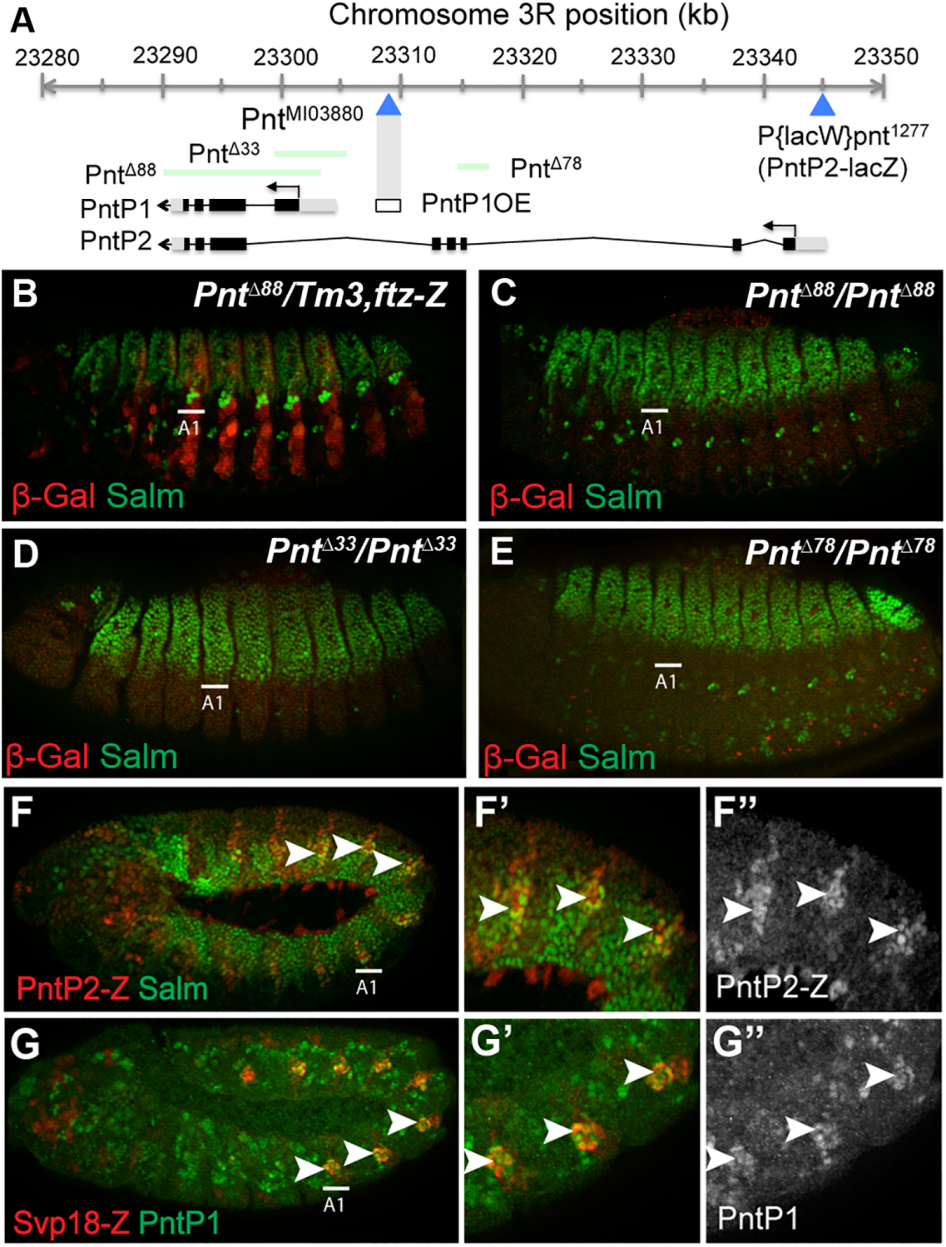
PntP1 and PntP2 are required for the specification of larval oenocytes. **Fig 1A:** Map of *pointed* locus showing the alternative promoters and transcripts that produce the *pntP1* and *pntP2* gene products as well as the deleted regions (green) that generate loss-of-function alleles for *pntP1* and *pntP2* (*pnt*^*Δ88*^), *pntP1* (*pnt*^*Δ33*^) and *pntP2* (*pnt*^*Δ78*^) [34, 36, 39]. The *pntP2-lacZ* (*P(lacW)pnt*^*1277*^ [40]) and *pnt*^*M103880*^ insertions are noted and the *PntP1OE* enhancer is highlighted. **Fig 1B–1E:** Lateral views of stage 15 *Drosophila* embryos with the following alleles: control *pnt*^*Δ88*^ heterozygotes (*pnt*^*Δ88*^*/Tm3*,*ftz-Z*) (B), *pnt*^*Δ88*^ homozygotes (C), *pnt*^*Δ33*^ homozygotes (D), and *pnt*^*Δ78*^ homozygotes (E). Embryos were immunostained for Spalt-major (Salm, green) and β-gal (red). The first abdominal segment (A1) is labeled for each embryo. Note clustered oenocytes (marked by high levels of Salm) only form in *pnt*^*Δ88*^ heterozygotes whereas the broader Salm expression pattern in the dorsal ectoderm is observed in all embryos. **Fig 1F:** Lateral view of *pntP2-lacZ Drosophila* embryo (stage 11) immunostained for Salm (green) and β-gal (red) reveals pntP2 activity in early oenocyte precursor cells (arrowheads). **Fig 1G:** Lateral view of *SvpΔ18-lacZ Drosophila* embryo (late stage 11) immunostained for PntP1 (green) and β -gal (red) reveals PntP1 expression in oenocyte precursor cells (arrowheads) shortly after their initial specification.

While genetic studies have shown that the EGF signaling pathway is required for larval oenocyte development, much less is known about the downstream effectors of oenocyte specification [21, 38]. To determine if the Pnt transcription factors are required for the development of these cells, we first analyzed embryos carrying a deletion within the *pnt* locus (*pnt*^*Δ88*^ [34]) that removes both *pntP1* and *pntP2* (**Fig 1A**). In contrast to wild type or *pnt*^*Δ88*^ heterozygote animals, *pnt*^*Δ88*^ null embryos lack oenocytes (marked by high Spalt-major (Salm) levels, **Fig 1B and 1C**). Analysis of embryos containing smaller deletions that only remove *pntP1* (*pnt*^*Δ33*^) or *pntP2* (*pnt*^*Δ78*^) [36, 39] similarly resulted in a complete loss of oenocytes, demonstrating that both *pnt* gene products are required to make this cell type (**Fig 1D and 1E**). Consistent with this finding, we found that both PntP1 and PntP2 are expressed in developing oenocytes. To analyze *pntP2* expression, we used a *PntP2-lacZ* line (*P(lacW)pnt*^*1277*^ [40]) and found β-gal expression in cells that include the early oenocyte precursors (**Fig 1F**). Similar analysis using a PntP1-specific antibody [41] revealed that PntP1 was also expressed in the developing oenocytes as marked by *SvpΔ18-lacZ*, albeit at a slightly later time point than *pntP2-lacZ* expression (**Fig 1G**). Thus, both *pnt* gene products are expressed within and required for the development of larval oenocytes during *Drosophila* embryogenesis.

**Fig 2:**
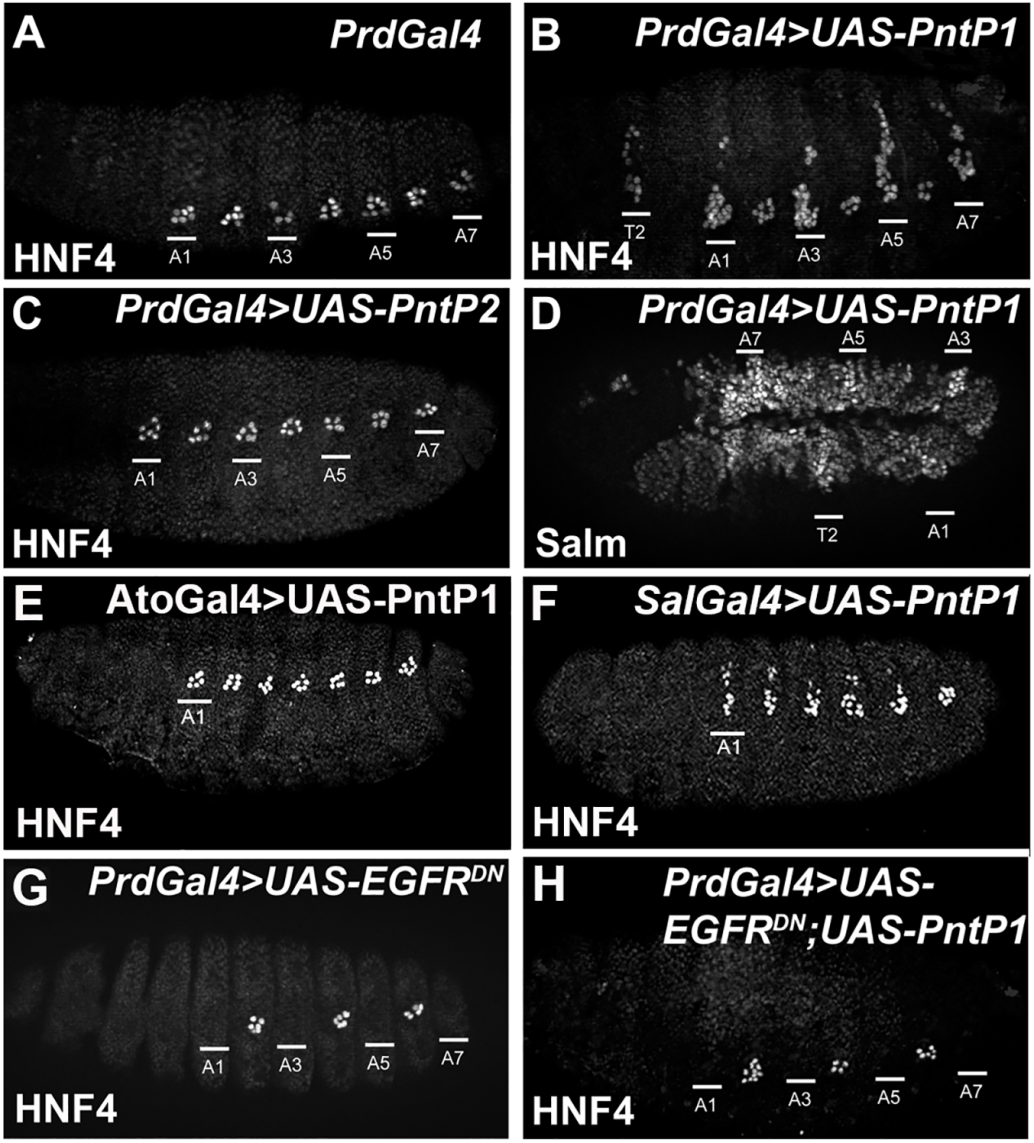
PntP1 can induce larval oenocyte fate in the embryonic dorsal ectoderm. **Fig 2A-2C:** Lateral views of control *PrdG4* (A), *PrdG4;UAS-PntP1* (B) and *PrdG4;UAS-PntP2* (C) embryos immunostained for the HNF4 oenocyte marker [42]. The PrdG4+ abdominal segments of each embryo are labeled, and the PrdG4+ second thoracic segment (T2) is labeled in the *PrdG4;UAS-PntP1* embryo (C). **Fig 2D:** Lateral view of *PrdG4;UAS-PntP1 Drosophila* embryo (stage 11) immunostained for Salm. The PrdG4+ thoracic (T2) and abdominal segments (A1/A3/A5/A7) are labeled and reveal increased Salm levels in the dorsal ectoderm. **Fig 2E and 2F:** Lateral views of *AtoG4;UAS-PntP1* (E) and *SpaltG4;UAS-PntP1* (F) embryos immunostained for the HNF4 oenocyte marker [42]. The PrdG4+ first abdominal segment (A1) of each embryo is labeled. **Fig 2G and 2H:** Lateral views of Stage 15 *PrdG4;UAS-EGFR*^*DN*^ (E) and *PrdG4;UAS- EGFR*^*DN*^*;UAS-PntP1* (F) embryos immunostained for HNF4 reveals the expression of PntP1 in absence of EGF signaling is unable to induce oenocyte formation. The PrdG4+ abdominal segments of each embryo are labeled.

To determine if the expression of each Pnt factor is sufficient to specify ectopic oenocytes, we used the *PrdG4* driver to express either *UAS-PntP1* or *UAS-PntP2* in every other segment of the *Drosophila* embryo. In this assay, only PntP1 was sufficient to induce ectopic oenocytes in PrdG4+ thoracic segments and extra oenocytes (HNF4+ cells) in PrdG4+ abdominal segments, whereas PntP2 had no effect on oenocyte specification (**Fig 2A–2C, S1 Fig**). Moreover, consistent with Spalt factors being significantly up-regulated by EGF signaling during larval oenocyte specification [23, 24], we found that PntP1 increases Salm expression levels in both thoracic and abdominal dorsal ectoderm cells in early embryos (**Fig 2D**). Since *PrdG4* is active in both the EGF sending (SOP) and receiving cells, we also tested the ability of PntP1 to induce oenocytes using Gal4 drivers expressed only within the SOP lineage (*AtoG4*) *versus* the surrounding ectoderm (*SpaltG4*) [22]. Consistent with PntP1 functioning cell autonomously within oenocyte precursors, we found that only *SpaltG4;UAS-PntP1* embryos produced ectopic oenocytes (**Fig 2E and 2F**). Overall, these findings suggest that the constitutively active PntP1 transcription factor is sufficient to induce oenocyte formation in the dorsal ectoderm, whereas PntP2 likely requires MAPK phosphorylation downstream of EGF signaling to activate gene expression and promote oenocyte cell fate [34, 36, 37].

Next, we wanted to determine if PntP1 is sufficient to induce oenocyte fate in the absence of EGF signaling. To address this question, we used *PrdG4* to express *UAS-PntP1* together with a dominant negative EGF receptor molecule (*UAS-EGFR*^*DN*^). Interestingly, expressing EGFR^DN^ on its own or with PntP1 resulted in a complete suppression of oenocyte specification (**Fig 2G and 2H**). Altogether, these findings suggest that while PntP1 is required to make oenocytes in the dorsal ectoderm, it is not sufficient to do so in the absence of EGF signaling. Thus, the EGF signaling pathway is required to regulate additional factors besides PntP1 to specify oenocyte fate.

### Identification of a PntP1 enhancer that activates gene expression in oenocyte precursor cells

**Fig 3:**
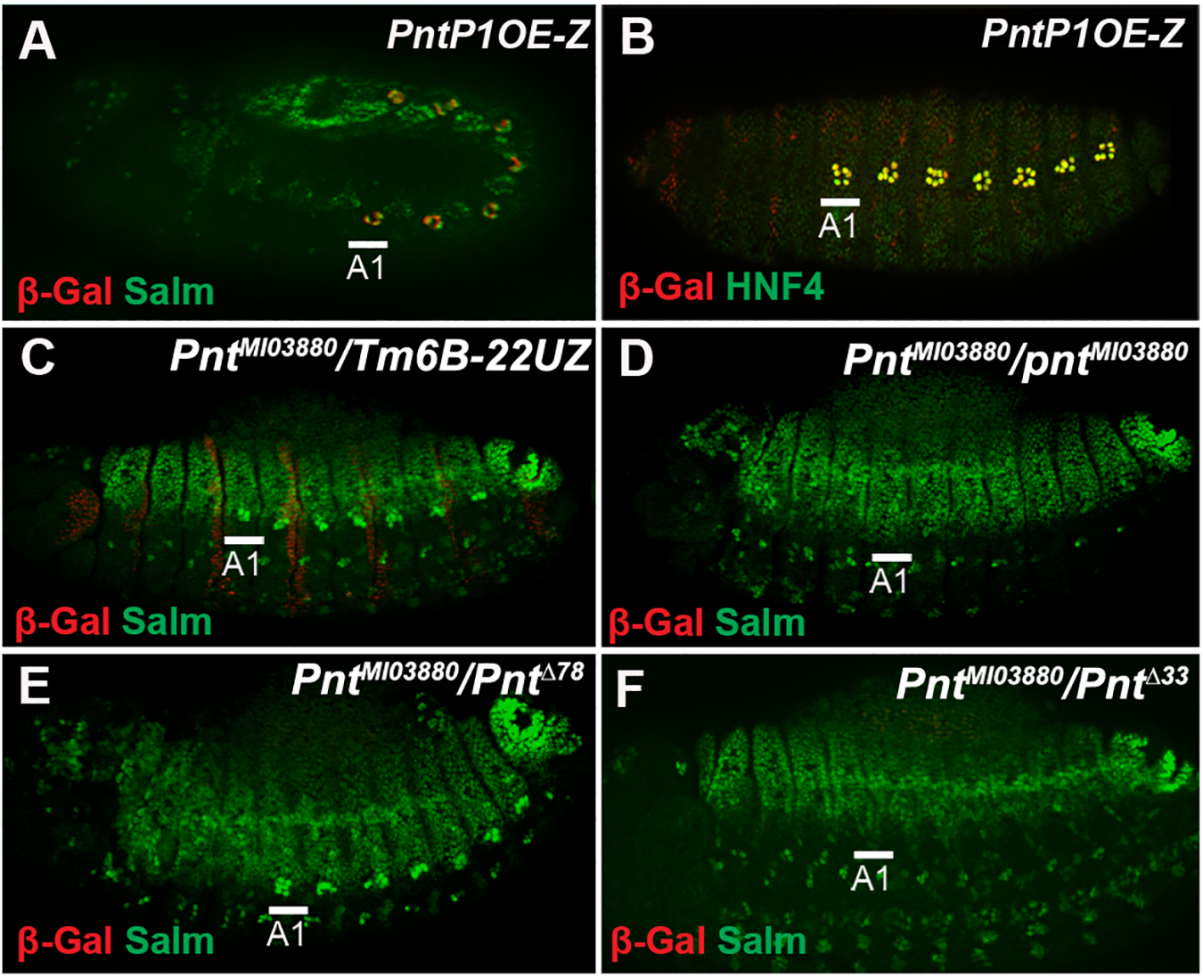
*PntP1OE* is an essential enhancer for the specification of oenocytes. **Fig 3A:** Lateral view of a *PntP1OE-lacZ* embryo (stage 11) immunostained for β-gal (red) and Salm (green) reveals enhancer activity in larval oenocyte precursor cells. **Fig 3B:** Lateral view of a *PntP1OE-lacZ* embryo (stage 15) immunostained for β-gal (red) and HNF4 (green) reveals enhancer activity in oenocytes. **Fig 3C and 3D:** Lateral view of a *pnt*^*MI03880*^*/TM6B-22uZ* heterozygote (C) and *pnt*^*MI03880*^ homozygote (D) embryo (stage 15) immunostained for β-gal (red) and Salm (green) reveals loss of oenocytes in *pnt*^*MI03880*^ mutant embryos. **Fig 3E and 3F:** Lateral view of *pnt*^*MI03880*^*/pnt*^*Δ78*^ (E) and *pnt*^*MI03880*^*/pnt*^*Δ33*^ (F) transheterozygote embryos (stage 15) immunostained for β-gal (red) and Salm (green) reveals that the *pnt*^*Δ78*^ (PntP2) null allele but not the *pnt*^*Δ33*^ (PntP1) null allele complements *pnt*^*MI03880*^.

While it has been established that EGF signaling activates the expression of *pntP1* at the transcriptional level, the *cis*-regulatory modules (CRMs) controlling *pntP1* expression are largely unknown. We identified a 700 base-pair sequence within the *pnt* locus (**Fig 1A**) that is sufficient to activate reporter expression (*PntP1OE-lacZ*) within oenocytes (**Fig 3A and 3B**). *PntP1OE-lacZ* expression is first detected in Salm+ oenocyte precursor cells during their specification, and *PntP1OE-lacZ* expression is highly enriched within mature HNF4-positive oenocytes of older embryos (**Fig 3A and 3B**). Interestingly, genomic analysis uncovered a fly line with a Minos transposable element (*pnt*^*MI03880*^) inserted within the *PntP1OE* enhancer of the *pnt* locus (**Fig 1A**). Embryos homozygous for this insertion lack oenocytes but retain *pntP1* expression in other regions of the *Drosophila* embryo such as the tracheal pits (**Fig 3C and 3D, S2 Fig**). To assess if *pnt*^*MI03880*^ selectively disrupts either *pntP1* or *pntP2* activity, we generated transheterozygote embryos that revealed the *pnt*^*MI03880*^ insertion complements the *pntP2* allele (*pnt*^*Δ78*^) but fails to complement the *pntP1* allele (*pnt*^*Δ33*^) (**Fig 3E and 3F**). Taken together, these findings strongly suggest that *PntP1OE* enhancer regulates *pntP1* expression in oenocyte precursor cells.

### EGF signaling and the Pnt transcription factors regulate *PntP1OE* enhancer activity in oenocyte precursors

**Fig 4:**
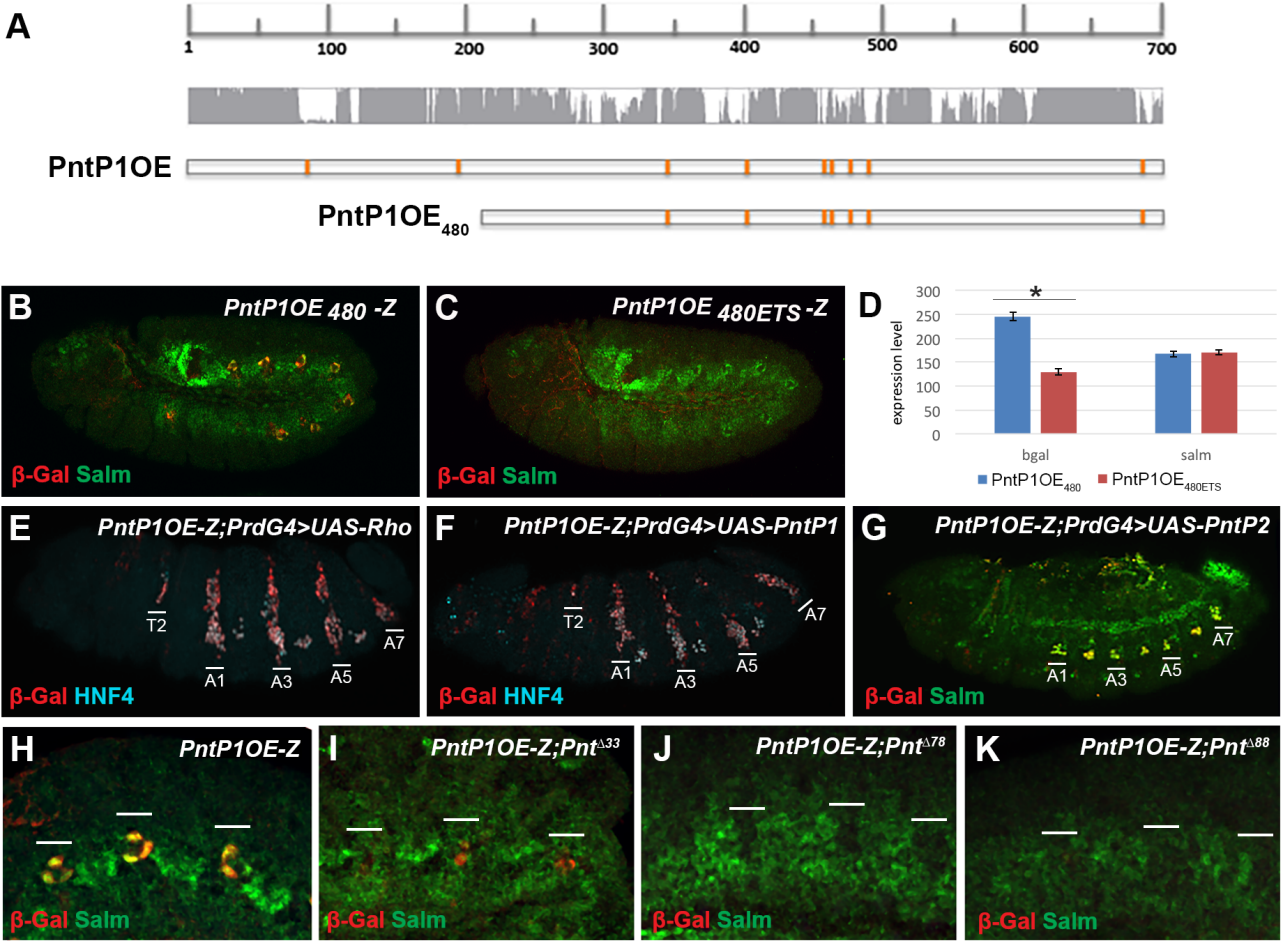
Pnt transcription factors directly regulate the *PntP1OE* enhancer in oenocyte precursor cells downstream of EGF signaling. **Fig 4A:** Sequence conservation of the 700 base-pair *PntP1OE* enhancer and the 480 base-pair region that contains seven ETS binding motifs (orange lines). The *PntP1OE*_*480*_ and *PntP1OE*_*480ETS*_ sequences tested in quantitative reporter assays are in **S3 Fig**. **Fig 4B and 4C:** Lateral views of *PntP1OE*_*480*_*-lacZ* (B) and *PntP1OE*_*480ETS*_*-lacZ* (C) embryos (stage 11) immunostained for β-gal and Salm (green). **Fig 4D:** Quantitation of *PntP1OE*_*480*_*-lacZ* and *PntP1OE*_*480ETS*_*-lacZ* reporter activity reveals a significant loss of enhancer activity when the seven ETS motifs are mutated. Spalt-major levels were measured as a control. * denotes p-value<0.01. **Fig 4E-4G:** Lateral views of *PrdG4;UAS-Rho* (E), *PrdG4;UAS-PntP1* (F) and *PrdG4;UAS-PntP2* (G) embryos immunostained for *PntP1OE-lacZ* activity (β-gal, red), and either HNF4 (blue) or Salm (green) as indicated. The PrdG4+ segments of each embryo were labeled and weaker thoracic (T2) induction of oenocytes were observed in *PrdG4;UAS-Rho* and *PrdG4;UAS-PntP1* embryos. Note, all HNF4-positive oenocytes express *PntP1OE-lacZ* but the levels of β-gal detected vary between cells (also see images in **S5 Fig** for example of variation in *PntP1OE-lacZ* activity in oenocytes). **Fig 4H and 4K:** Close-up lateral views of stage 11 *PntP1OE-lacZ* abdominal segments with the following alleles: wild type (H), *pnt*^*Δ33*^ homozygotes (I), *pnt*^*Δ78*^ homozygotes (J) and *pnt*^*Δ88*^ homozygotes (K). Embryos were immunostained for Salm (green) and β-gal (red). Note the lack of Salm up-regulation in all *pnt* mutants, and that only the *pntP1* mutant (I) expresses low levels of *PntP1OE-lacZ*.

The expression of *pntP1* is often induced by receptor tyrosine kinase (RTK) signaling in *Drosophila* via relatively unknown mechanisms. To determine if *PntP1OE* is directly regulated by EGF signaling in oenocyte precursors, we first analyzed the *PntP1OE* sequence and identified seven conserved ETS binding motifs within a 480 base pair region (*PntP1OE*_*480*_, **Fig 4A**). Transgenic reporter analysis of *PntP1OE*_*480*_*-lacZ* reveals it is sufficient to activate gene expression in oenocyte precursors, and mutation of all seven ETS sequences (*PntP1OE*_*480ETS*_*-lacZ)* results in a significant loss of reporter activity (**Fig 4B–4D,** see **S3 Fig** for *OE*_*480*_ and *OE*_*480ETS*_ sequences). These findings are consistent with Pnt transcription factors playing a direct role in enhancer activation downstream of EGF signaling. Congruent with this idea, we found that activation of the EGF pathway via ectopic expression of *rho* (*PrdG4;UAS-rho*) or *PntP1* (*PrdG4;PntP1)* was sufficient to induce *PntP1OE-lacZ* activity (**Fig 4E and 4F**). In contrast, expression of PntP2 (*PrdG4*;*UAS-PntP2*), which requires an EGF-dependent phosphorylation event for activity, did not significantly alter *PntP1OE-lacZ* activity (**Fig 4G**).

To determine if both PntP1 and PntP2 are required for *PntP1OE-lacZ* activation in oenocyte precursors, we next analyzed embryos mutant for both *pntP1* and *pntP2* (pntΔ88), *pntP1* alone (pntΔ33), or *pntP2* alone (pntΔ78). In all cases, we observed a dramatic loss of *PntP1OE-lacZ* expression in older embryos (**S4 Fig**). However, analysis of *PntP1OE-lacZ* activity in early embryos revealed that *pntP1* mutants expressed weak β-gal levels (**Fig 4H and 4I**), whereas mutants that lacked *pntP2* (either alone or in combination with *pntP1*) showed no early *PntP1OE-lacZ* activity (**Fig 4J and 4K**). These findings are consistent with an EGF signal activating the PntP2 protein via phosphorylation and thereby stimulating the *PntP1OE* enhancer to induce PntP1 expression. Once activated, PntP1 can then positively auto-regulate itself. Hence, in the absence of *pntP2*, *PntP1OE-lacZ* expression is not observed, and no PntP1 protein is produced. In contrast, without *pntP1*, PntP2 induces an initial up-regulation of *PntP1OE-lacZ* expression during active EGF signaling, but its expression is not maintained. Moreover, while *pntP1* mutants can weakly induce *PntP1OE-lacZ expression*, these cells fail to significantly up-regulate Spalt expression and develop into oenocytes (compare **Fig 4H and 4I**). Thus, PntP1 and PntP2 are required to both upregulate *PntP1OE* and induce oenocytes.

### The Abdominal-A Hox factor and Homothorax enhance oenocyte specification downstream of EGF signaling

**Fig 5:**
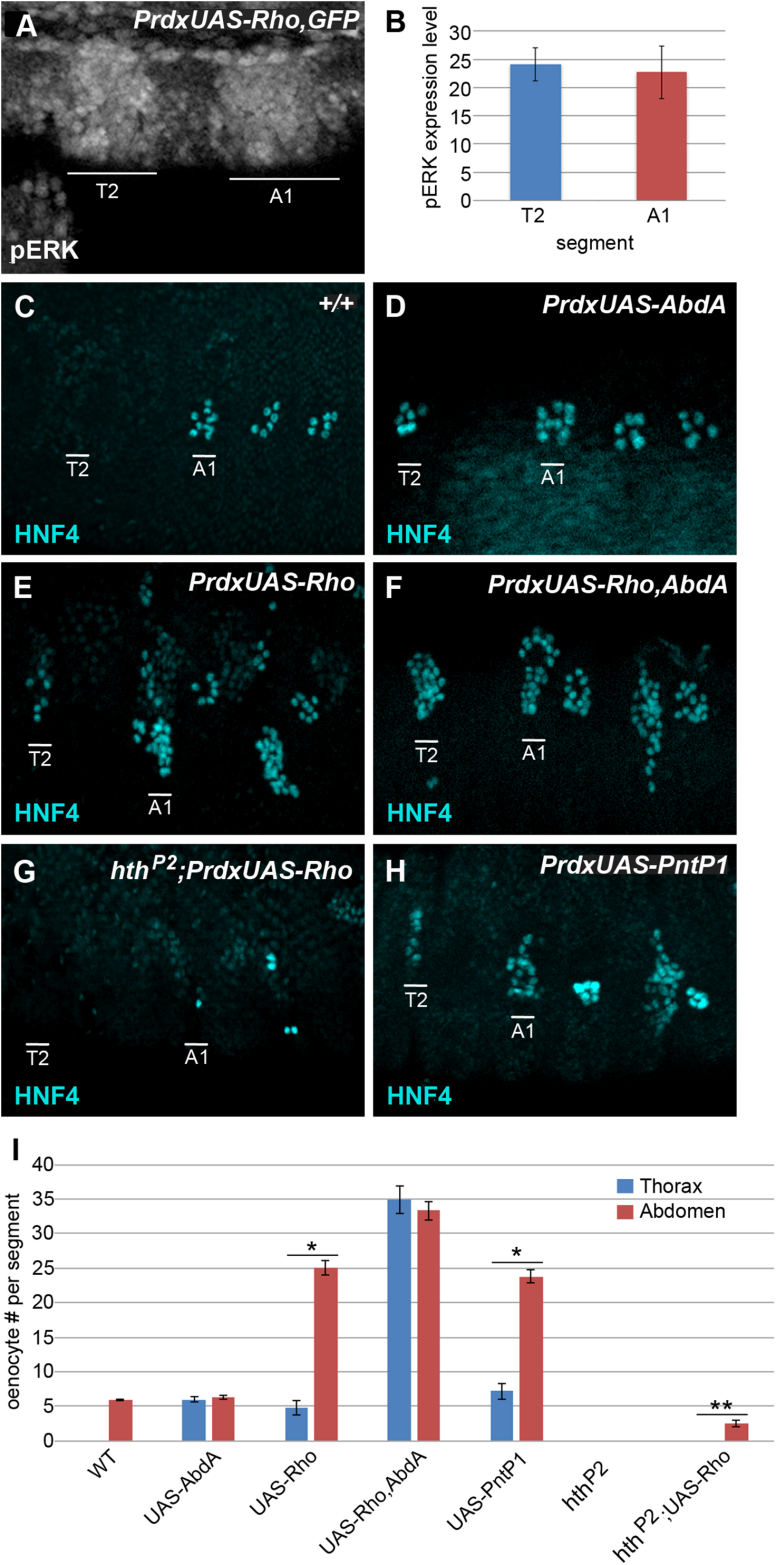
Abd-A and Hth function enhance oenocyte formation in both a cell-autonomous and non-cell-autonomous manner. **Fig 5A:** Lateral view of the thoracic and first abdominal segments of a *PrdG4;UAS-Rho* embryo (stage 11) immunostained for phospho-ERK. The PrdG4-active second thoracic (T2) and first abdominal (A1) segments are labeled. **Fig 5B:** Quantitation of phospho-ERK expression in the PrdG4+ thoracic and abdominal segments of *PrdG4;UAS-Rho* embryos reveals equal activity between segments. **Fig 5C-5F:** Lateral views of wild type (C), *PrdG4;UAS-AbdA* (D), *PrdG4;UAS-Rho* (E), and *PrdG4;UAS-Rho;UAS-AbdA* (F) embryos (stage 15) immunostained for HNF4 (blue). The PrdG4+ thoracic (T2) and abdominal (A1) segments are labeled. Note, oenocyte numbers in wild type animals can vary from four to ten per abdominal segment [21, 24, 26]. Quantitative assessment of significant changes in oenocyte numbers are reported in **Fig 5I**. **Fig 5G:** Lateral view of *hth*^*p2*^*;PrdG4;UAS-Rho* embryo (stage 15) immunostained for HNF4 (blue). The PrdG4+ thoracic (T2) and abdominal (A1) segments are labeled. **Fig 5H:** Lateral view of *PrdG4;UAS-PntP1* embryo (stage 15) immunostained for HNF4 (blue). The PrdG4+ thoracic (T2) and abdominal (A1) segments are labeled. **Fig 5I:** Quantitation of thoracic and abdominal oenocyte number per segment of at least 8 embryos from each genetic background. Red bars represent number of oenocytes per PrdG4+ abdominal segment, whereas blue bars represent number of oenocytes per PrdG4+ thoracic segment. * denotes p-value<0.01 and ** denotes p-value<0.05 for thoracic and abdominal segments.

The current model of larval oenocyte specification is that an Abd-A Hox complex with Extradenticle (Exd) and Homothorax (Hth) is required to induce an EGF signal by direct activation of the *rhomboid* (*rho*) serine protease in the abdominal C1 sensory organ precursor (SOP) cells [28, 38]. The neighboring cells receive the EGF signal and activate the Pnt transcription factors to initiate the specification of larval oenocyte fate. If Abd-A is only non-cell autonomously required to activate *rho* to induce oenocyte fate, we reasoned that equal expression of *rho* in the thorax will bypass the requirement for Abd-A in making oenocytes. To test this idea, we first established that the *PrdG4* driver can be used to drive equal expression of Rho (*UAS-Rho;PrdG4*) and thereby EGFR activation within thoracic and abdominal segments as evidenced by equivalent activity of phospho-ERK (**Fig 5A and 5B**) [43]. Consistent with previous studies [22], we found that thoracic expression of Rho was sufficient to induce oenocytes, indicating Abd-A is not strictly required for oenocyte development. However, comparisons between thoracic and abdominal segments revealed dramatic differences in the number of oenocytes specified with nearly six times as many made in abdominal segments than in the thorax (**Fig 5E and 5I**). Importantly, co-expression of Abd-A with Rho (*PrdG4;UAS-Rho;UAS-Abd-A*) stimulated equal numbers of oenocytes in both thoracic and abdominal segments (**Fig 5F and 5I**). Moreover, the expression of Abd-A alone is sufficient to induce the same number of oenocytes (approximately 6) in the thorax and the abdomen (**Fig 5D and 5I**). Altogether, these findings suggest that while both thoracic and abdominal cells are equally capable of receiving the EGF signal (equal phospho-Erk levels), the abdominal cells are more highly competent to become oenocytes in an AbdA-dependent manner.

Next, we wanted to assess the role of Hth, a Hox co-factor in oenocyte specification. As mentioned above, Hth forms a direct complex with Abd-A on a *rho* cis-regulatory module to stimulate gene expression in abdominal SOPs [28, 38]. Loss-of-function genetic studies revealed that oenoctyes fail to form in a strong *hth* hypomorph (*hth*^*P2*^, **Fig 5I**) [44]. To determine if the loss of oenocytes is simply due to a failure to activate *rho* expression and thereby EGF secretion, we used *PrdG4* to express Rho in the absence of *hth* function (*UAS-rho;PrdG4*,*hth*^*P2*^) and found that no oenocytes were induced by high levels of Rho expression in the thorax and very few oenocytes were induced in the abdomen of *hth* mutant embryos (**Fig 5G and 5I**). Thus, these findings demonstrate that like Abd-A, Hth function is not only essential for activating *rho* in SOPs but it also greatly enhances the specification of oenocytes downstream of EGF signaling.

### An Abdominal-A Hox complex regulates PntP1OE activity in oenocyte precursor cells

**Fig 6:**
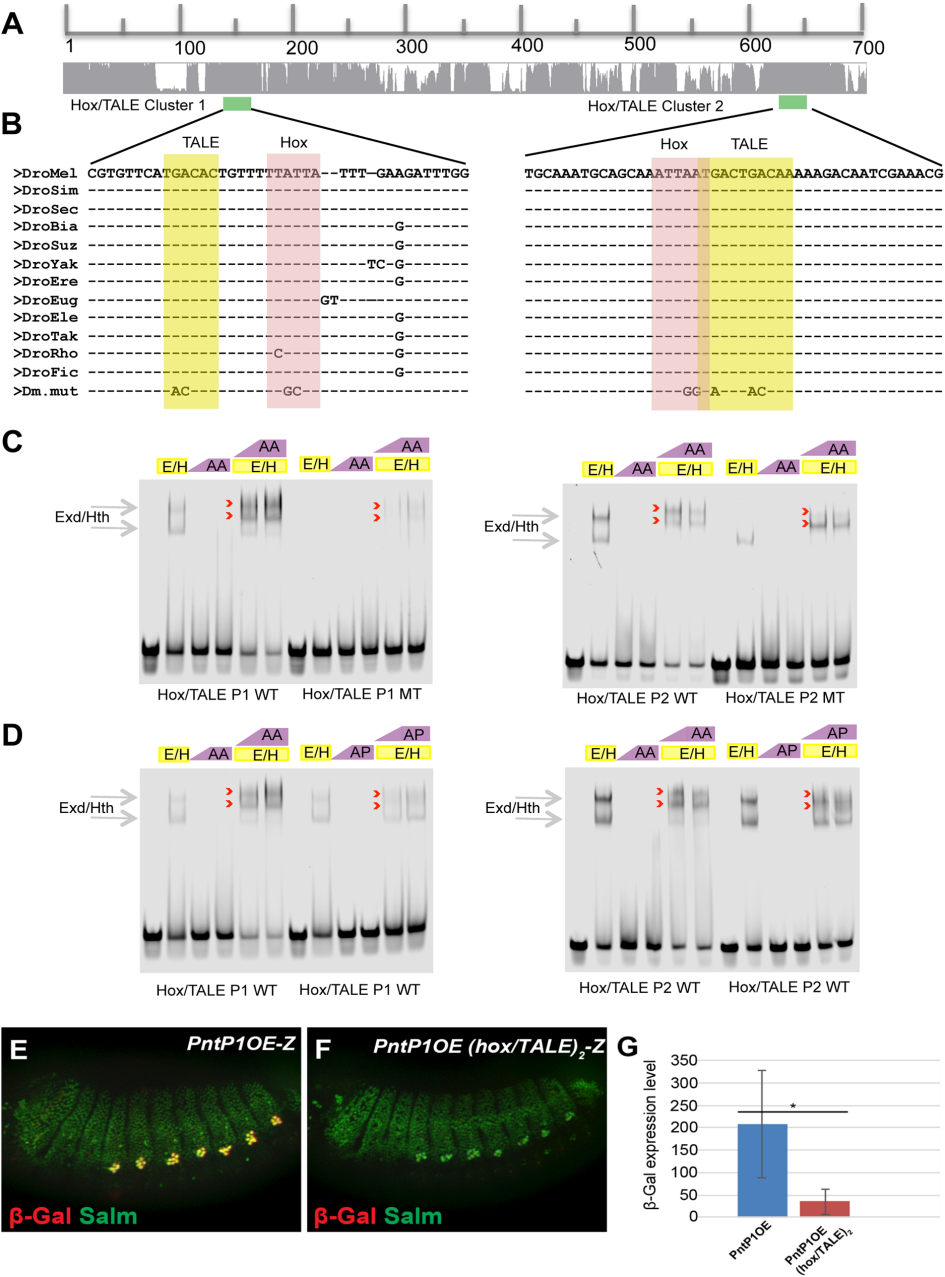
The *PntP1OE* enhancer is regulated by an Abdominal-A Hox complex. **Fig 6A and 6B:** Sequence conservation of the 700 base-pair *PntP1OE* enhancer. The location of the two regions containing the highly conserved Hox (pink) and TALE (yellow) binding sites are shaded and the sequence mutations tested in EMSAs and quantitative reporter assays are noted. **Fig 6C:** DNA binding assays using purified Abd-A and Exd/Hth proteins and wild type and mutant Hox/TALE probes. Gray arrows denote Exd/Hth complex formation on DNA, whereas red arrowheads denote Exd/Hth/Abd-A complex formation on DNA. Note, diminished DNA binding activities on mutant probes. **Fig 6D**: Comparisons in DNA binding activity and complex formation with Exd/Hth between equimolar concentrations of purified Abd-A (AA) and the Antp (AN) thoracic Hox factor. Gray arrows denote Exd/Hth complex formation on DNA, whereas red arrowheads denote Exd/Hth/Hox complexes on DNA. Note, diminished DNA binding activity of Antp compared to Abd-A. **Fig 6E and 6F:** Lateral views of *PntP1OE-lacZ* (G), and *PntP1-OE(hox/TALE)*_*2*_*-lacZ* (H) embryos (stage 15) immunostained for β-gal and HNF4 (blue). **Fig 6G:** Quantitation of *PntP1OE-lacZ* and *PntP1OE(hox/TALE)*_*2*_*-lacZ* reporter activity reveals a significant loss of enhancer activity when the Hox/TALE motifs are mutated. * denotes p-value<0.01.

The ability of Abd-A to enhance the competency of cells to commit to an oenocyte fate downstream of EGF signaling suggests that this abdominal Hox factor regulates target genes in the oenocyte specification pathway such as the *PntP1OE enhancer*. Consistent with this idea, we found that *PntP1OE-lacZ* expression was induced by ectopic Abd-A (*PrdG4;UAS-AbdA*) in the thorax (**S5 Fig**). Moreover, sequence analysis of *PntP1OE* revealed two regions with conserved binding sites for Hox and the Three Amino-acid Loop Extension (TALE) homeodomain factors that include Hth and Exd (**Fig 6A and 6B**). Electromobility Shift Assays (EMSAs) using purified Exd, Hth, and Abd-A proteins reveals these proteins bind probes containing each site and point mutations within these sites greatly decrease complex formation (**Fig 6C**). In contrast, equimolar concentrations of the thoracic Hox factor Antennapedia (Antp) binds these probes with significantly less affinity, consistent with Abd-A selectively enhancing the competency of oenocyte specification (**Fig 6D**). To determine if these sites are essential for proper *PntP1OE-lacZ* regulation in oenocytes, we generated transgenes containing Hox/Exd/Hth binding site mutations within both regions and found a significant loss of oenocyte enhancer activity (**Fig 6E–6G**).

Altogether, these data are consistent with an Abd-A Hox complex enhancing oenocyte competence by potentiating the EGF signal in the signal receiving oenocyte precursor cells. If *pntP1* is the only target gene required to be activated by Abd-A to promote oenocyte formation, we reasoned that ectopic expression of PntP1 using *PrdG4* (*PrdG4;UAS-PntP1*) should induce as many oenocytes in the thorax as the abdomen. However, we found that like *rho*, PntP1 expression is not sufficient to induce as many oenocytes in thoracic segments that lack Abd-A expression compared to the Abd-A expressing abdominal segments (see **Fig 2B** and **Fig 5H and 5I**). These findings suggest that Abd-A is likely to enhance oenocyte competency by regulating additional downstream target genes and not just through the potentiation of the EGF signal via *pntP1* activation.

## Discussion

**Fig 7:**
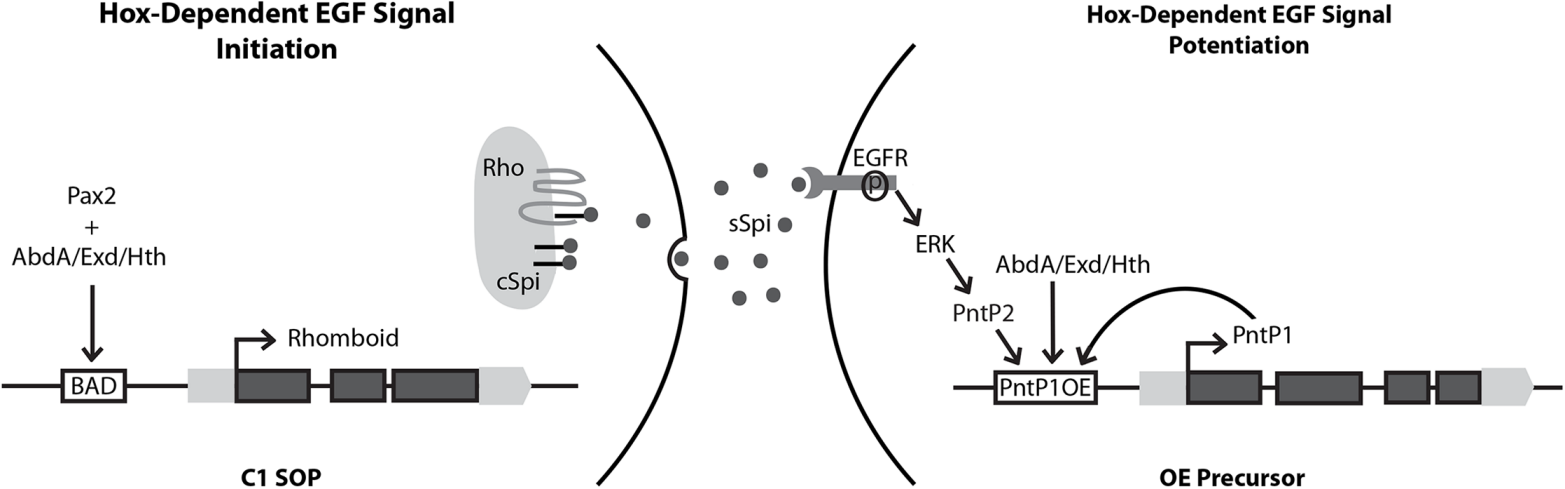
Model of oenocyte specification induced by Abd-A dependent EGFR signaling derived from abdominal C1 SOP. Schematic representation of the Hox-dependent initiation and potentiation of the EGF signal. An Abd-A/Hth/Exd complex together with the *Drosophila* Pax2 transcription factor activates *rhomboid* gene expression through direct binding of the RhoBAD enhancer to promote the cleavage and secretion of the Spitz EGF ligand. The secreted EGF ligand (sSpitz) binds the EGFR of neighboring cells and thereby activates a kinase signaling pathway to activate PntP2. PntP2 subsequently activates the *PntP1OE* enhancer to induce *pntP1* expression. Once expressed, PntP1 can also regulate *PntP1OE* to form a positive autoregulatory loop. The Abd-A Hox complex also contributes to the potentiation of the EGF signal via the direct activation of the *PntP1OE* enhancer.

In this study, we investigated how a key component of the anterior-posterior specification pathway (the Abd-A Hox factor) regulates the Epidermal Growth Factor (EGF) signaling pathway to promote formation of hepatocyte-like cells (oenocytes). Prior studies had shown that Abd-A is non-cell autonomously required to promote oenocyte fate by directly activating a gene (*rhomboid*) that causes the secretion of an EGF ligand (Spitz) from a subset of abdominal sensory organ precursor (SOP) cells [22, 27, 28]. Here, we show that Abd-A also plays a cell autonomous role in promoting oenocyte fate by directly regulating the transcriptional expression of the PntP1 ETS transcription factor (**Fig 7**). Since the activation of Pnt transcription factors lies downstream of EGF signaling [35], these findings suggest that Abd-A potentiates the EGF signal to promote oenocyte fate. Below, we discuss how these data provide new insights into both the mechanisms by which the Hox and EGF signaling pathways intersect as well as the gene regulatory networks that are required for oenocyte specification.

The EGF signaling pathway largely regulates the specification of cell types by regulating the expression of downstream target genes via ETS transcription factors [35]. *Drosophila* has three ETS transcription factors regulated by the EGF pathway. The PntP1 and PntP2 ETS proteins largely function as transcriptional activators that are produced via alternative promoters, whereas the Yan ETS factor functions as a transcriptional repressor to thereby antagonize the EGF pathway [33, 34, 36, 37, 45]. Of these three proteins, both Yan and PntP2 are directly modified by the MAPK pathway. PntP2 requires MAPK phosphorylation to activate transcription, while MAPK phosphorylation of Yan results in its nuclear export and degradation. In contrast, the PntP1 ETS factor is regulated by RTK signaling at the transcriptional level, and once expressed, PntP1 does not require post-translational modifications to activate gene expression. Moreover, at least in the *Drosophila* pupal eye, the half-life of the PntP1 protein can be as long as 6 hours [32]. Thus, EGF signaling transiently promotes target gene expression via post-translational modifications of two ETS factors and the transcriptional induction of PntP1 results in a longer lasting effect on target gene expression.

Consistent with Pnt factors being key effectors of the EGF pathway, we found that both PntP1 and PntP2 are required to specify larval oenocytes in the *Drosophila* embryo. Together with previous studies showing that loss-of-function *yan* mutations result in the formation of extra abdominal oenocytes (~12 per cluster compared to ~6 per cluster in wild type embryos [26]), these findings support the following model: Abd-A/Hth/Exd complexes activate the expression of the *rhomboid* protease to trigger EGF ligand secretion from the C1 abdominal SOP cells [27, 28]. The neighboring dorsal ectoderm cells receive the transient EGF signal, stimulate the MAPK signal transduction pathway, and thereby activate PntP2 and inactivate Yan [24, 25, 45]. Activated PntP2 as well as the Abd-A/Hth/Exd complexes bind the *PntP1OE* enhancer to activate *PntP1* transcription within oenocyte precursors (**Fig 7**). Once expressed, the constitutively active PntP1 protein can positively auto-regulate itself to further potentiate the EGF signal and promote oenocyte specification. However, it should be noted that if the EGF signal is of sufficient strength, the cell autonomous requirement of the Abd-A Hox factor for oenocyte specification can be bypassed. For example, direct activation of *rho* in the thorax can induce oenocytes, but to a much lesser extent than in *abd-A* expressing segments (**Fig 5**). Taken together with previous publications [22, 28], these studies demonstrate that an Abd-A/Hth/Exd transcription factor complex contributes to oenocyte development in two ways: direct activation of the EGF signal within the C1 SOP and direct potentiation of the EGF signal in the receiving cell by activation of *pntP1* during oenocyte differentiation (**Fig 7**).

Two additional findings from our studies suggest that further integration of EGF and Abd-A Hox factors are likely to occur during oenocyte specification beyond the activation of *pntP1*. First, our studies reveal that EGF signaling must regulate additional factors required for the specification of oenocyte fate as the expression of high levels of PntP1 is not sufficient to induce oenocytes when the EGF signal is inhibited using a dominant negative protein (**Fig 2H**). Second, Abd-A must also regulate additional factors in oenocyte precursors besides PntP1, as the expression of PntP1 in thoracic segments is unable to induce as many oenocytes compared with abdominal segments that express Abd-A (**Fig 5H and 5I**). While it is currently unclear which additional genes are targeted by these factors during oenocyte specification, candidate factors that have been previously implicated to lie downstream of EGF signaling in oenocyte development include the Spalt-major (Salm) zinc finger protein, the Seven-up (Svp) and Hepatocyte Nuclear Factor 4 (HNF4) hormone receptors, and the ventral-veinless (Vvl) POU homeodomain protein [22, 24, 26].

Several fundamental questions arise from these studies such as how many oenocyte target genes are regulated by the Abd-A Hox and Pnt factors, and how are these factors integrated to regulate target gene expression during oenocyte development? Studies focused on other Hox regulated morphological structures and cell types suggest Hox factors commonly regulate many targets within a gene regulatory network [8, 9, 11]. For example, studies on how Ultrabithorax (Ubx) promotes haltere development and how Abdominal-B (Abd-B) specifies genitalia in *Drosophila* reveal that Hox factors are likely to regulate numerous target genes throughout the network [19, 20, 46]. Moreover, while we currently do not know how Abd-A and the Pnt transcription factors are physically integrated, a recent study using bimolecular fluorescence complementation (BiFC) found that Abd-A and Pnt can interact in *Drosophila* embryos [47]. Hence, future studies focused on the transcriptional mechanisms used by Abd-A and Pnt factors is likely to shed new insight into the underlying gene regulatory networks required for the development of these hepatocyte-like cells.

## Materials and methods

### Plasmid and transgenic fly generation

The *PntP1OE cis*-regulatory element (3R: 23309267…23309966 R6.13) was amplified by PCR from genomic DNA, and *PntP1OE* mutations and deletions were introduced by PCR mediated mutagenesis. All enhancers were cloned into the *pLacZ-attB* plasmid and confirmed by DNA sequencing. Transgenic fly lines were generated by ΦC31 integration into the 51C insertion site [48] (Injections by Rainbow Transgenics).

### *Drosophila* stocks and embryo staining

The following fly lines were used: *PrdGal4*, *AtoGal4*, *SpaltGal4*, *hth*^*P2*^, *UAS-Abd-A*, *UAS-PntP1*, *UAS-PntP2*, *UAS EGFR*^*DN*^, *UAS-Rho*, *PntP2-lacZ*, *SvpΔ18-lacZ*, *pnt*^*Δ88*^, *pnt*^*Δ78*^, *pnt*^*Δ33*^, and *pnt*^*MI03880*^. Embryos were collected, fixed and immuno-stained using standard procedures at 25°C. The following primary antibodies were used: Abd-A (guinea pig 1:500) [28], HNF4 (rat 1:500) [49]; Salm (rabbit 1:500) [50]; PntP1 (rabbit 1:500) [41]; pERK (mouse 1:50) and β-gal (chicken 1:1000) (Abcam). Images were taken on an apotome-configured Zeiss fluorescent microscope. Oenocyte numbers were quantified using HNF4 positive staining. Imaris64 software was used to measure the expression level of wild type *PntP1OE-lacZ* versus *PntP1OE*(*hox-hth*)_2_*-lacZ* and the wild type *PntP1OE*_*480*_*-lacZ* versus *PntP1OE*_*480ETS*_*-lacZ* inserted in the same locus. Oenocytes were identified and counted by positive HNF4 staining in stage 15 or older embryos. Age matched embryos were fixed, immunostained, and imaged under identical conditions to quantify β-gal and HNF4 levels in oenocytes. Analysis was conducted on samples whose HNF4 staining was not significantly different. Each data set was comprised of a minimum of 10 embryos. A T-test was used to determine significance.

### Protein purification and EMSAs

The following proteins were purified from BL21 bacterial cells as previously described [51]: His-tagged Abd-A; His-Antp; His-Hth and untagged Exd heterodimers [52, 53]. DNA probes were annealed from primers labeled with IRdye700 at 5`terminus [54]. EMSAs were performed as previously described using native polyacrylimide gel electrophoresis. The acrylamide gels were imaged using a Odyssey LiCOR cLX scanner.

## Supporting information

S1 Fig Normal oenocyte formation in abdominal segments ectopically expressing PntP2. Quantitation of oenocyte numbers in PrdG4+ abdominal segments of control and PntP2 expressing embryos (N = at least 24 segments per genetic condition).
(TIF)

S2 Fig The *Pnt*^*MI03880*^ insertion does not disrupt Pntp1 expression in trachael pits. Lateral view of stage 11 *Pnt*^*MI03880*^*/Tm6B-22UZ* (A) and *Pnt*^*MI03880*^*/Pnt*^*MI03880*^ (B) embryos immunostained for β-gal (red) and PntP1 (green) reveals PntP1 expression in tracheal pits (TP). First abdominal segment (A1) of each embryo is labeled.
(TIF)

S3 Fig Sequence of PntP1OE480 and PntP1OE480_ETS_ enhancers tested in transgenic reporter assays. PntP1OE480 and PntP1OE480_ETS_ sequences with conserved wild type ETS motifs shown in red (A) and mutant ETS motifs shown in blue (B).
(TIF)

S4 Fig *PntP1OE-lacZ* expression is lost in *pnt* mutant embryos. Lateral views of stage 15 wild type (A), *pnt*^*Δ33*^*/pnt*^*Δ33*^ (B), *pnt*^*Δ78*^*/pnt*^*Δ78*^ (C), and *pnt*^*Δ88*^*/pnt*^*Δ88*^ (D) embryos immunostained for *PntP1OE-lacZ* activity (β-gal, red) and Salm (green). First abdominal segment (A1) is labeled. Note, *pnt* mutant embryos lack oenocytes and the only significant β-gal expression is in the dorsal ectoderm (arrowhead).
(TIF)

S5 Fig The Abd-A Hox factor can induce oenocytes and *PntP1OE-lacZ* activity in the thorax. **A)** Lateral view of stage 15 *PrdG4;UAS-AbdA* embryo immunostained for *PntP1OE-lacZ* activity (β-gal, red) and HNF4 (green). The PrdG4+ thoracic (T2) and first abdominal segment (A1) are labeled. Note, oenocytes and *PntP1OE-lacZ* activity are induced in the thorax. **B)** Close-up view of the segments reveals that all of the oenocytes express β-gal protein (white) but vary in intensity.
(TIF)
