# Supplementary figures and images for: A Hox complex activates and potentiates the Epidermal Growth Factor signaling pathway to specify Drosophila oenocytes
Source: PLoS Genet. 2017 Jul 17;13(7):e1006910. doi: 10.1371/journal.pgen.1006910 (PMC5536354; doi:10.1371/journal.pgen.1006910)

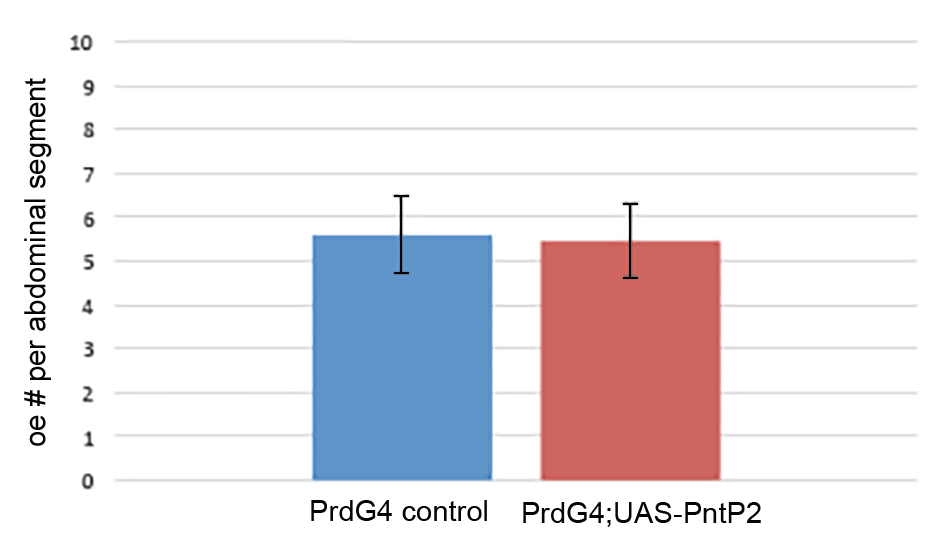

Supplement: S1 Fig — Quantitation of oenocyte numbers in PrdG4+ abdominal segments of control and PntP2 expressing embryos (N = at least 24 segments per genetic condition). (TIF) [file pgen.1006910.s001.tif]

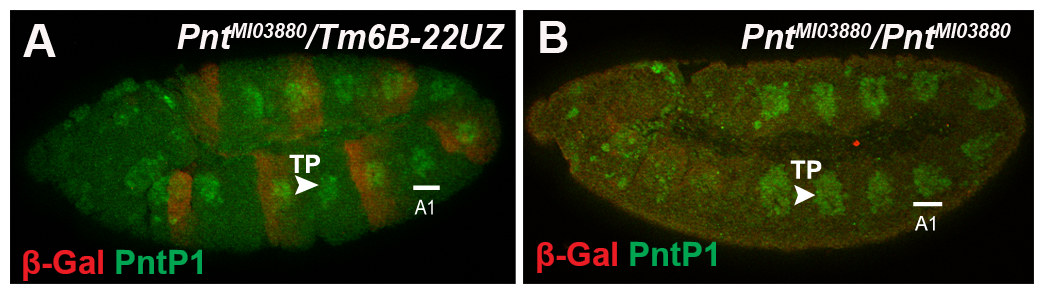

Supplement: S2 Fig — Lateral view of stage 11 PntMI03880/Tm6B-22UZ (A) and PntMI03880/PntMI03880 (B) embryos immunostained for β-gal (red) and PntP1 (green) reveals PntP1 expression in tracheal pits (TP). First abdominal segment (A1) of each embryo is labeled. (TIF) [file pgen.1006910.s002.tif]

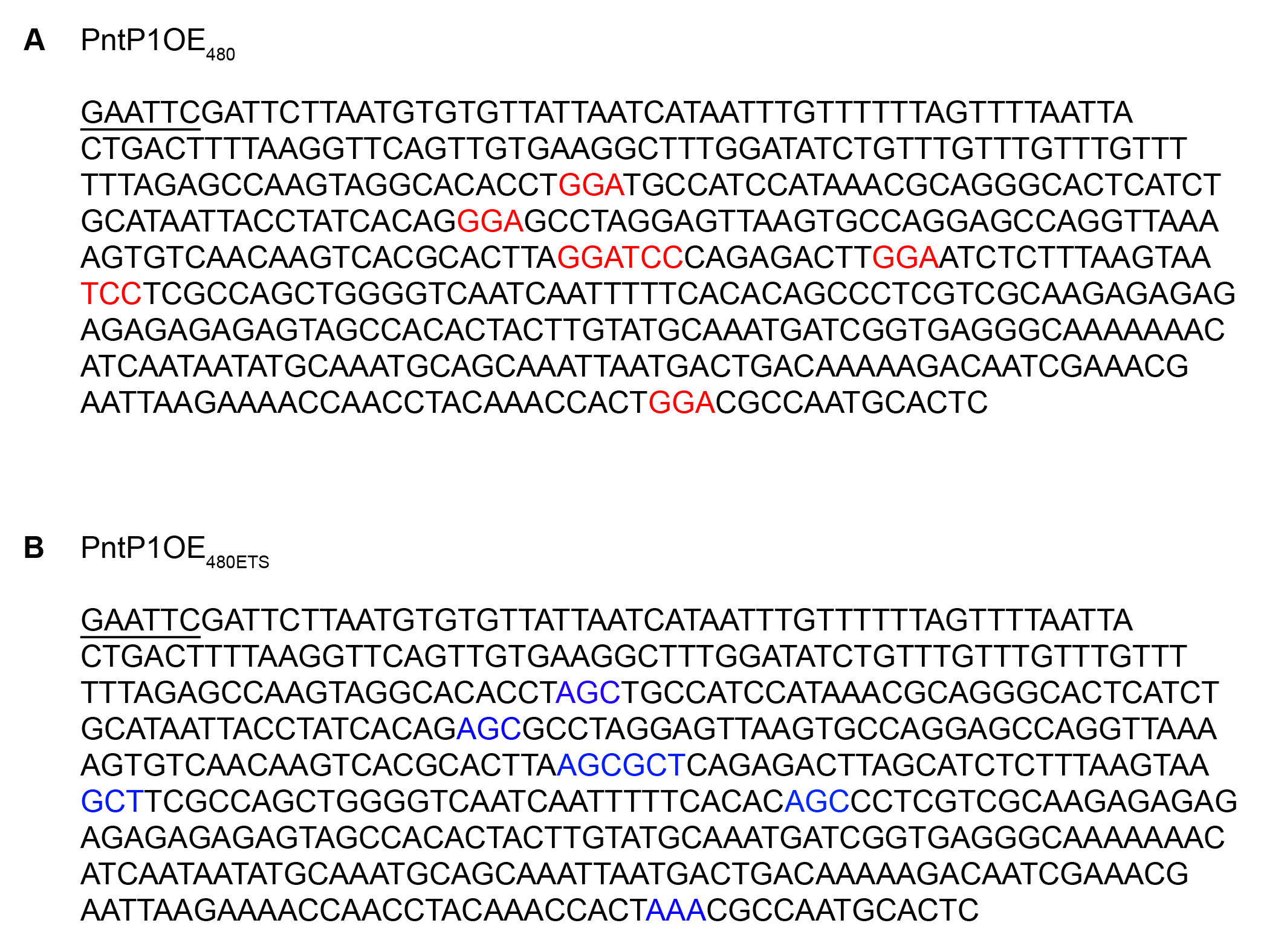

Supplement: S3 Fig — PntP1OE480 and PntP1OE480ETS sequences with conserved wild type ETS motifs shown in red (A) and mutant ETS motifs shown in blue (B). (TIF) [file pgen.1006910.s003.tif]

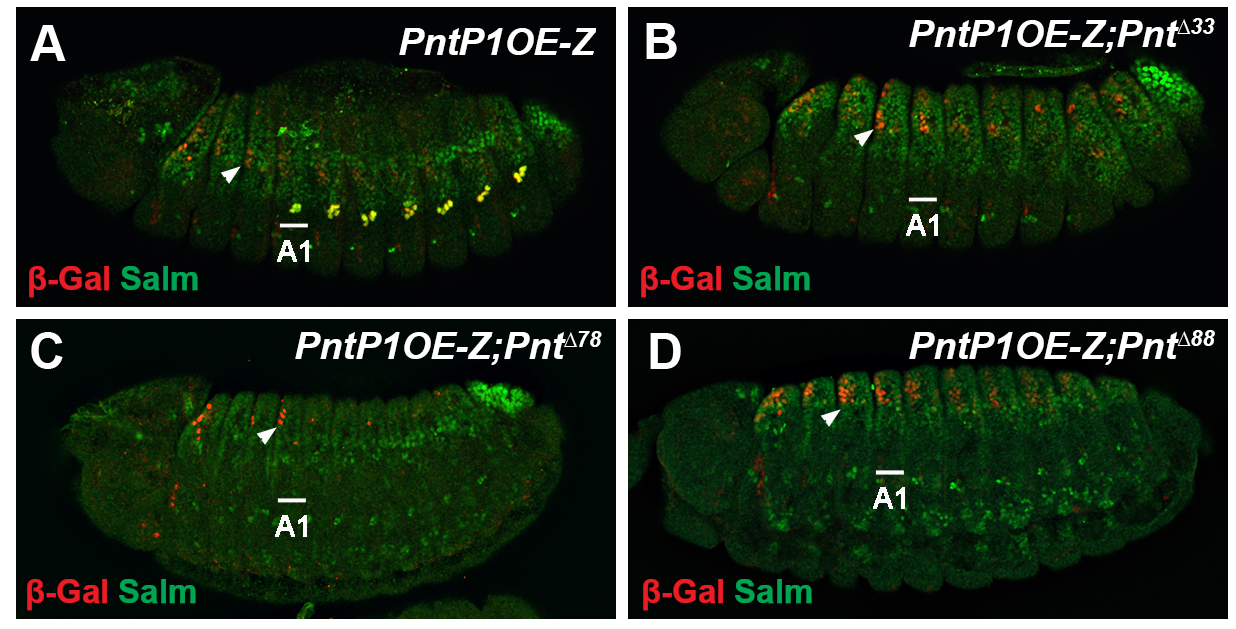

Supplement: S4 Fig — Lateral views of stage 15 wild type (A), pntΔ33/pntΔ33 (B), pntΔ78/pntΔ78 (C), and pntΔ88/pntΔ88 (D) embryos immunostained for PntP1OE-lacZ activity (β-gal, red) and Salm (green). First abdominal segment (A1) is labeled. Note, pnt mutant embryos lack oenocytes and the only significant β-gal expression is in the dorsal ectoderm (arrowhead). (TIF) [file pgen.1006910.s004.tif]

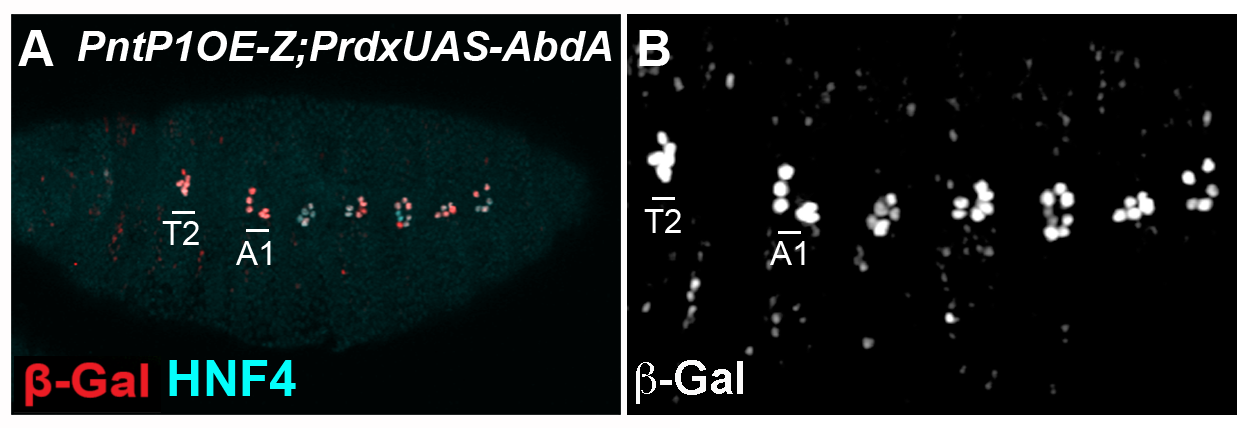

Supplement: S5 Fig — A) Lateral view of stage 15 PrdG4;UAS-AbdA embryo immunostained for PntP1OE-lacZ activity (β-gal, red) and HNF4 (green). The PrdG4+ thoracic (T2) and first abdominal segment (A1) are labeled. Note, oenocytes and PntP1OE-lacZ activity are induced in the thorax. B) Close-up view of the segments reveals that all of the oenocytes express β-gal protein (white) but vary in intensity. (TIF) [file pgen.1006910.s005.tif]
